# Copper-Epoxy Interface Engineering for High-Frequency Chip-to-Chip Interconnects

**DOI:** 10.1149/2162-8777/ae170a

**Published:** 2025-11-04

**Authors:** Junghyun Park, Ignace Agbadan, Monsuru Dauda, Mustapha Bello, John Hendershot, Soundarzo Tasnim, Omotolani Oduyebo, Sunggook Park, Anthony Engler, Jaimal Williamson, Varughese Mathew, Cigdem Atalay-Oral, John Flake

**Affiliations:** 1 Gordon A. and Mary Cain Department of Chemical Engineering, Louisiana State University, Baton Rouge, Louisiana 70803, United States of America; 2 Department of Mechanical & Industrial Engineering and Center for Bio-Modular Multiscale Systems, Louisiana State University, Baton Rouge, Louisiana 70803, United States of America; 3 Texas Instruments Incorporated, Dallas, Texas 75243, United States of America; 4 NXP Semiconductors, Austin, Texas 78735, United States of America

**Keywords:** oxidation, multichip package, polymer-metal adhesion, metal interlayer, cladding, coupling agent, Chip-to-chip (C2C) interconnect

## Abstract

The reliability of high-frequency chip-to-chip (C2C) interconnects requires the durability of Cu interconnects and polymer dielectric interfaces. In this study, we examine the temperature cycling performance of Cu-epoxy interfaces, including methylimidazole and amine functional silane adhesion promoters along with electroplated or electroless metal interlayers such as Ni, NiCo_0.64_P_0.25_, NiW_0.48_P_0.10_, and CoW_0.66_P_0.17_. X-ray photoelectron spectroscopy is used to determine chemical states and compositions. O diffusion is analyzed using energy-dispersive X-ray spectroscopy. The adhesion strength was measured using a peel tester, and sheet resistance was characterized using a four-point probe before and after temperature cycle tests. Insertion losses were measured using a vector network analyzer as a function of temperature cycling. Our results indicate that organic adhesion promoters such as silanes show improvements in the initial adhesion; however, extended temperature cycling results in weakened interfaces associated with Cu oxidation. The CoW_0.66_P_0.17_ metallic interlayer is more durable and exhibits a minimal 0.02 dB mm^−1^ change in insertion losses at 18 GHz after 1500 cycles.

## Highlights

- The Cu-epoxy interface performance is studied over 1500 temperature cycles.
- The temperature stability of organic and metal interlayers is studied for Cu-epoxy.
- Organic adhesion promoters, such as silane and azole, improve initial adhesion.
- Electroless CoW_0.66_P_0.17_ interface enhances adhesion due to its stable oxides.
- The CoW_0.66_P_0.17_ shows stability with a 0.02 dB mm^−1^ S21 change after 1500 cycles.

## Graphical abstract

**Figure:**
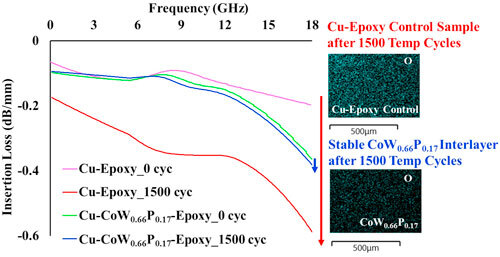

Multichip packages for artificial intelligence (AI) and autonomous vehicle applications require high-frequency chip-to-chip (C2C) interconnects capable of data transfer rates over 1.8 TB/s per GPU or 50 GB/s per interconnect.^1–8^ Extending 2.5D and 3D packaging to these rates relies on the mechanical and signal integrity of Cu interconnects and polymer dielectrics such as epoxies;^1^ however, the trade-off between performance and reliability presents a challenge.^9,10^ Recent reports have shown greater insertion losses at higher frequencies; for example, insertion losses exceeding 0.8 dB were reported up to 200 GHz and 1.5 dB up to 300 GHz.^11–13^ Power losses are attributed to skin effect, which concentrates the current near the surface and is exacerbated by surface roughness.^14^ Thus, smooth interconnects are desirable for signal integrity;^15,16^ however, the smooth interconnects compromise mechanical reliability, such as adhesion strength.^10,17^

Previous works have shown that adhesion at Cu-epoxy interfaces may be improved by the deposition of adhesion promoters such as coordination compounds,^18–21^ coupling agents,^22–24^ or metal interlayers.^25^ Organic coordination compounds such as azoles and thiols enhance chemisorption on Cu surfaces through their lone-pair electrons and by mitigating corrosion.^18–21,26^ However, directly adding organics like azoles to epoxies causes other reactions with the epoxies, their entrapment within the epoxy networks, or evaporation during the thermal curing process.^27^ Other coupling agents, such as silanes, are known to provide covalent metal-O-Si bonds between Cu and epoxy, enhancing chemical adhesion and improving the mechanical integrity of Cu-epoxy interfaces.^22–24^ As noted by Nambafu et al., dipodal silanes, which contain more hydrolyzable silanol groups, also enhance hydrolytic stability;^28,29^ however, the long-term reliability of silane adhesion promoters is limited.^30^ Ultimately, equilibrium thermodynamics and the inherent reactivity of Cu, including oxidation reactions with water and O, result in their limited stability.^31^

Metal interlayers like CoWP, NiWP, or other such interlayers enhance adhesion by creating metal mixtures or alloys with Cu (e.g., Cu-Co) and can provide more stable metal oxides (such as WOx) at the interface with epoxy.^32,33^ The metal interlayers, including their relatively more stable oxides (compared to brittle Cu oxides), have been studied for inter-die interconnects in back-end-of-line (BEOL) integrations to reduce electromigration;^34–36^ however, their relatively higher resistivities compared to Cu also lead to higher signal losses because of increased resistance.^37^

This study investigates organic adhesion promoters, including azole coordination compounds and silane coupling agents, as well as metal interlayers, which influence the mechanical, electrical, and chemical durability of Cu-epoxy interfaces. The engineered interfaces include: methylimidazole and amine functional silane adhesion promoters, along with metal interlayers such as Ni, NiCo_0.64_P_0.25_, NiW_0.48_P_0.10_, and CoW_0.66_P_0.17_. Temperature cycle tests (TCT) were performed for 1,500 cycles, with temperatures ranging from −40 to 125 °C. X-ray photoelectron spectroscopy (XPS) is used to determine chemical states and compositions of organic adhesion promoters and metal interlayers. The oxidation process at the Cu-epoxy interfaces is investigated using energy-dispersive X-ray spectroscopy (EDS). Mechanical and signal integrity were assessed using a peel tester, a four-point probe, and a vector network analyzer (VNA) to gain fundamental insights into how material and interface design influence the reliability of C2C interconnects.

## Experimental

### Cu-Epoxy joint formation

0.01 mm-thick 99.9% Cu polycrystalline foils, obtained from MilliporeSigma, USA, were cleaned with 10% hydrochloric acid (HCl) for 10 min before surface treatments. The cleaned Cu surface has a root-mean-square roughness of 45 ± 11 nm.^30^ Surface treatments were applied to cleaned Cu foils before molding epoxy. These treatments included azole coating, silane coating, Ni electroplating, or electroless plating with NiCo_0.64_P_0.25_, NiW_0.48_P_0.10_, or CoW_0.66_P_0.17_. Epoxy blends comprised 52% diglycidyl ether of bisphenol-A epoxy resin (DGEBA, EPON 828, Miller-Stephenson Chemicals, USA), 47% methyl-5-norbornene-2,3-dicarboxylic anhydride (Nadic Methyl Anhydride, NMA, Electron Microscopy Sciences, USA), and 1% imidazole (Sigma-Aldrich, USA). Epoxy mixture was poured onto Cu foils after organic or metal coating and cured for 8 h at 115 °C.^10,38^ During the curing process, an isopropyl segment is formed by opening the cyclic ether ring of epoxides in the DGEBA resin and connecting to NMA.^10^

### Azole and silane coating

Azole and silane coatings were applied to cleaned Cu foils before molding epoxy. For azole coating, Cu foils were dipped into a 3 mM aqueous solution of 2-methylimidazole (azole) for 1 min. Azole-coated Cu foils were heated at 150 °C for 30 min. For silane coating, Cu foils were dipped into a 0.3 M solution of 3-aminopropyltrimethoxysilane (silane) for 5 min. The solvent of the silane solution consisted of 20% deionized water and 80% ethanol. Silane-coated Cu foils were heated at 90 °C for 15 min.

### Ni, NiCoP, NiWP, and CoWP plating

Ni electroplating and electroless plating of NiCo_0.64_P_0.25_, NiW_0.48_P_0.10_, or CoW_0.66_P_0.17_ were performed on cleaned Cu foils before molding epoxy. Ni layer was electroplated at 10 mA cm^−2^ for 1 min. The Ni electroplating bath contains 1.14 M NiSO_4_‧6H_2_O, 0.68 M NaCl, and 0.78 M H_3_BO_3_, with a pH of 5. Electroless plating of NiCo_0.64_P_0.25_, NiW_0.48_P_0.10_, or CoW_0.66_P_0.17_ was performed on Cu foils for 1 h. NiCo_0.64_P_0.25_ bath consisted of 0.20 M NaH_2_PO_2_‧H_2_O, 0.17 M Na_3_C_6_H_5_O_7_‧2H_2_O, 0.23 M (NH_4_)_2_SO_4_,0.10 M NiSO_4_‧6H_2_O, and 0.04 M CoSO_4_‧7H_2_O. pH was adjusted to 8.5–9 with NH_4_OH. The bath temperature was maintained at 60 °C. NiW_0.48_P_0.10_ bath consisted of 0.20 M NaH_2_PO_2_‧H_2_O, 0.17 M Na_3_C_6_H_5_O_7_‧2H_2_O, 0.23 M (NH_4_)_2_SO_4_,0.10 M NiSO_4_‧6H_2_O, and 0.06 M Na_2_WO_4_‧2H_2_O. pH was adjusted to 8.5–9 with NH_4_OH. The bath temperature was maintained at 85 °C. CoW_0.66_P_0.17_ bath consisted of 0.20 M NaH_2_PO_2_‧H_2_O, 0.17 M Na_3_C_6_H_5_O_7_‧2H_2_O, 0.23 M (NH_4_)_2_SO_4_, 0.04 M CoSO_4_‧7H_2_O, and 0.06 M Na_2_WO_4_‧2H_2_O. pH was adjusted to 8.5–9 with NH_4_OH. The bath temperature was maintained at 90 °C.

Metal interlayer thickness was measured using cross-sectional images obtained from scanning electron microscopy. (SEM, ThermoScientific Helios G5 CXe Plasma FIB/SEM, USA). The film compositions were determined by XPS (Scienta Omicron ESCA 2SR X-ray Photoelectron Spectroscope, Sweden). XPS was carried out on Cu surfaces before molding epoxy. A monochromatic Al Kα (1486.7 eV) was used at a power of 300 W (15 kV) with a pass energy of 30 eV, dwell time of 1 s, and 0.5 eV step.

### 90° peel test

**Figure 1.**
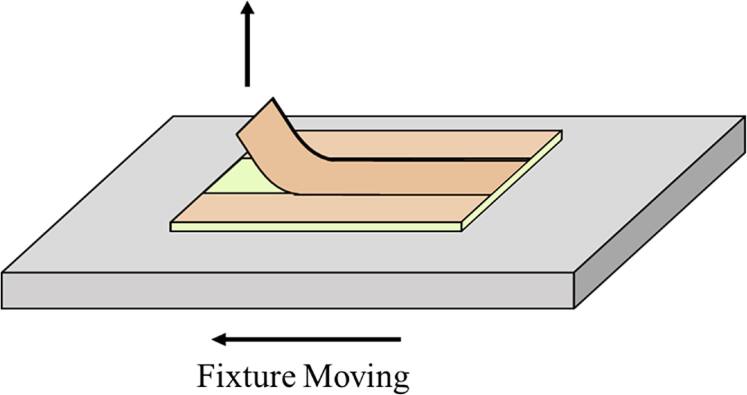
Schematic image of 90° peel tests.

For the 90° peel test, a utility knife was used to provide a 10 mm-wide Cu strip for a 90° peel test as presented in Fig. S1. As shown in Fig. 1, the Cu strip was peeled from the epoxy at a rate of 25 mm min^−1^ following ASTM standards B-533.^39^

### Four-point probe and VNA

Signal integrity was investigated using a four-point probe and a VNA. The effect of TCT on sheet resistance was investigated by four-point probe (Ossila, UK). The sheet resistance was measured on the Cu surface of the Cu-epoxy systems. For the VNA, microstrips were fabricated using conventional lithography and etching. A thin layer of S1813 photoresist was spin-coated onto the samples at 3000 rpm for 45 s, then heated for 1 min at 115 °C.^40^ The S1813 photoresist was exposed to 365 nm light.^41^ Then, a 351 developer was used on the surface of the photoresist. Finally, the samples were etched with 2.45 M CuCl_2_·2H_2_O and 6.18 M HCl, resulting in a Cu microstrip with dimensions of 76 mm in length, 1 mm in width, and 10 μm in height interconnects as shown in Fig. S2.^10,42^ As shown in Fig. S2a, SMA connectors were soldered to Cu transmission line before measuring the s-parameters with a vector network analyzer (VNA, Copper Mountain, USA). Soldered SMA connectors were linked to SMA cables and SMA adapters of the VNA, as presented in Fig. S2b. Prior to measuring the s-parameters, the connectors were de-embedded using the S2 VNA software.^43^

### TCT

TCT was carried out within a temperature range of −40 °C to 125 °C, following the JESD22-A104E standard.^44^ The temperature cycle was performed 1500 times. The relative humidity in the temperature cycling chamber is 60% at 25 °C.

O diffusion was examined using EDS on Cu surfaces after the peel test of the Cu and molded epoxy systems. The samples were stored in a vacuum chamber to mitigate further oxidation.

## Results and Discussion

### Thin film characterization

**Figure 2.**
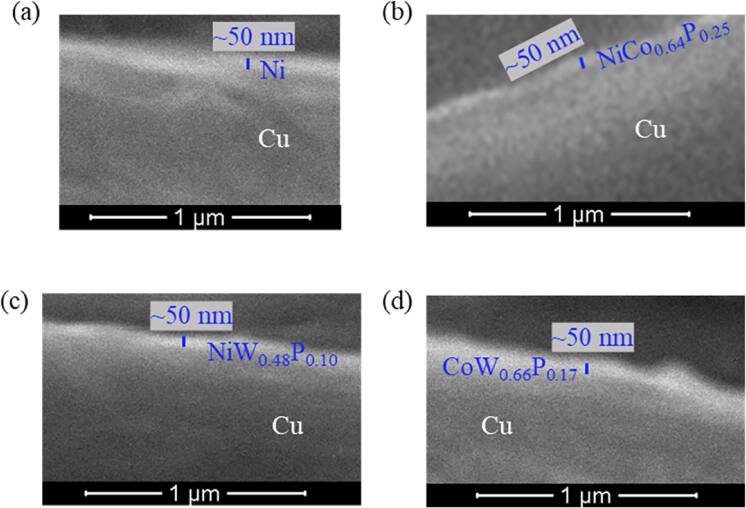
Cross-sectional SEM images of Cu with (a) Ni, (b) NiCo_0.64_P_0.25_, (c) NiW_0.48_P_0.10_, and (d) CoW_0.66_P_0.17_. SEM images were obtained before molding the epoxy.

Figure 2 shows cross-sectional SEM images of Cu with metal interlayers, including Ni, NiCo_0.64_P_0.25_, NiW_0.48_P_0.10_, and CoW_0.66_P_0.17_. SEM was conducted after metal plating on Cu and before epoxy molding. Metal interlayers have approximately 50 nm thickness.

**Figure 3.**
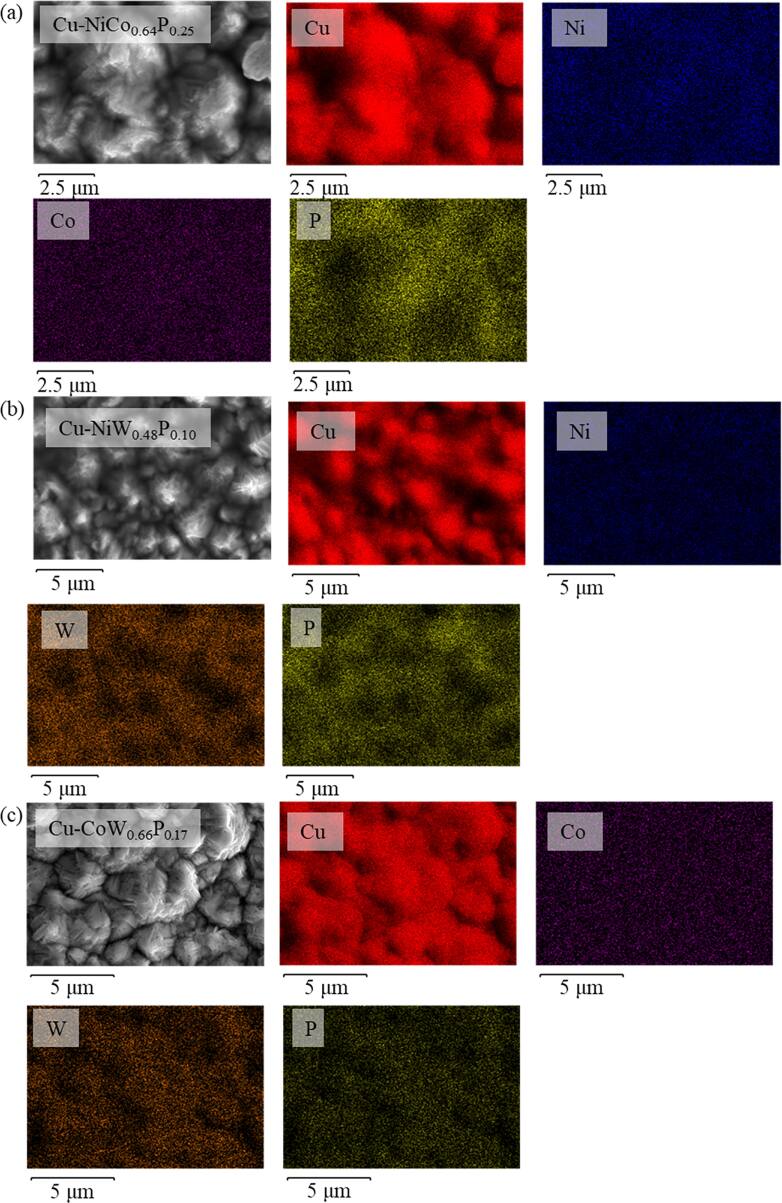
EDS-SEM images after electroless plating of (a) NiCo_0.64_P_0.25_, (b) NiW_0.48_P_0.10_, and (c) CoW_0.66_P_0.17_ on Cu. EDS images were obtained before molding epoxy.

EDS-SEM provides elemental analysis for thin films. Figures S3 and 3a–3c show the EDS mapping of Cu surface after surface modification with methylimidazole, amine functional silane, and metal interlayers such as Ni, NiCo_0.64_P_0.25_, NiW_0.48_P_0.10_, and CoW_0.66_P_0.17_. Figure 3 exhibits that the metal elements are homogeneously distributed in each sample; Fig. 3a shows Ni, Co, and P, Fig. 3b presents Ni, W, and P, Fig. 3c shows Co, W, and P on Cu.

**Figure 4.**
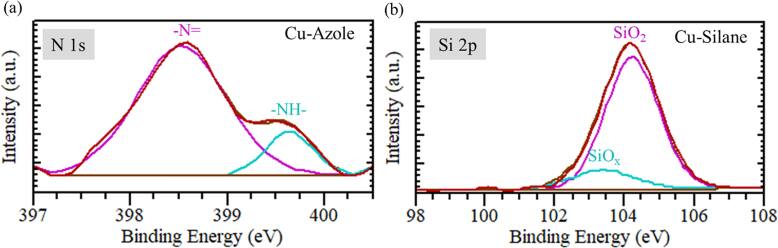
XPS spectra of (a) N 1 s for Cu coated with 2-methylimidazole (azole) and (b) Si 2p for Cu coated with 3-aminopropyltrimethoxysilane (silane) before the epoxy was molded.

Figure 4 presents N 1 s and Si 2p XPS spectra for Cu with azole and silane coating. Figure 4a shows a peak at ∼399 eV, corresponding to imine nitrogen (=N-) in the ring or the N in the deprotonated azole ring.^45,46^ A peak at ∼400 eV corresponds to protonated nitrogen (-NH-) in the azole ring.^45,46^ Figure 4b presents 103 and 104 eV attributed to SiO_x_ sub-oxides and a surface terminated with SiO_2_ (Si^4+^).^47,48^

**Figure 5.**
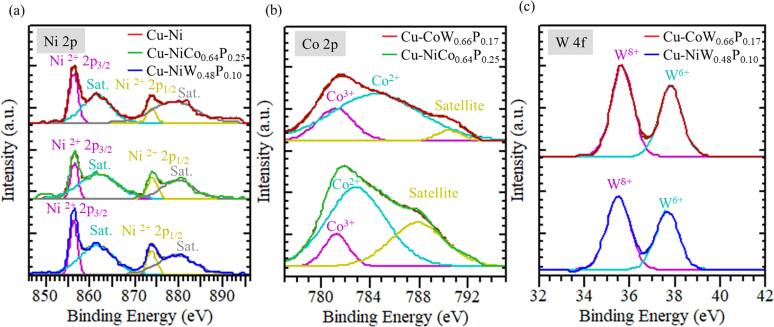
XPS spectra of (a) Ni 2p, (b) Co 2p, and (c) W 4 f from Cu surfaces after plating with Ni, NiCo_0.64_P_0.25_, NiW_0.48_P_0.10_, and CoW_0.66_P_0.17_. XPS spectra were obtained before the epoxy was molded.

The compositions of the alloy are determined using XPS and are as follows: NiCo_0.64_P_0.25_, NiW_0.48_P_0.10_, and CoW_0.66_P_0.17_. XPS was performed on the Cu surface after electroless plating of Cu foils and before epoxy molding. Figure 5a shows Ni^2+^ 2p_3/2_ (856 eV), Ni^2+^ 2p_1/2_ (874 eV), and satellites at 861 ± 1 and 879 ± 1 eV for Cu plated with Ni, NiCo_0.64_P_0.25_, and NiW_0.48_P_0.10_.^49^ Figure 5b shows the Co 2p spectra of XPS performed on Cu surfaces with electroless-plated Co. The peaks at 781 eV and 783 ± 1 eV, observed on the Cu surfaces with NiCo_0.64_P_0.25_ and CoW_0.66_P_0.17_, correspond to Co^3+^ and Co^2+^ in the Co 2p_3/2_ region, respectively,^50^ indicating that Co^3+^ and Co^2+^ coexist. The peak centered at 789 ± 1 eV is a satellite peak.^51^ Figure 5c presents the W 4 f XPS of Cu surfaces with electroless-deposited W. The peaks at 36 eV and 38 eV are attributed to W 4f_5/2_ and W 4f_7/2_, respectively.^51^ The W 4f_5/2_ peak is associated with W^*δ*+^ in NiW_0.48_P_0.10_ and CoW_0.66_P_0.17_.^51^ The W 4f_7/2_ peak at 38 eV is associated with W^6+^ species such as WO_3_.^51^

### 90° peel test

**Figure 6.**
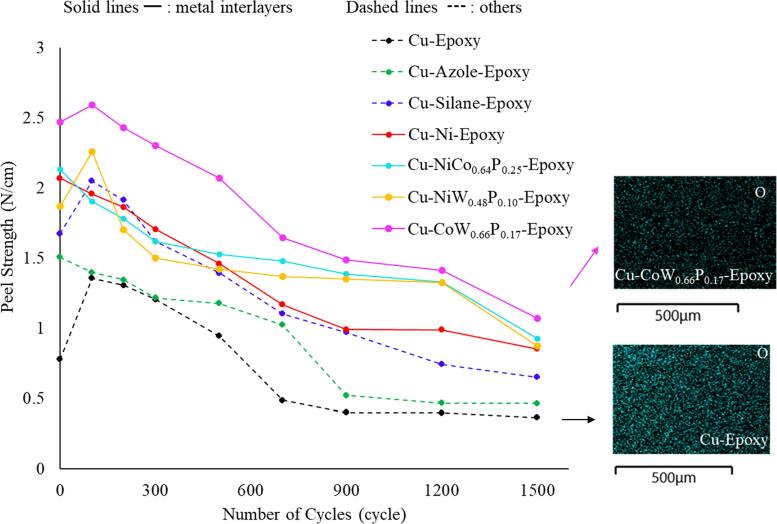
Peel strength of Cu and epoxy systems with azole coating, silane coating, Ni electroplating, and electroless plating of NiCo_0.64_P_0.25_, NiW_0.48_P_0.10_, and CoW_0.66_P_0.17_ after TCT. Solid lines represent metal interlayers, and dashed lines indicate the others. The two images on the right side present EDS mapping of Cu surfaces, exhibiting O diffusion at the Cu-epoxy interface with/without the CoW_0.66_P_0.17_ interlayer after 1500 temperature cycles.

Figure 6 presents the peel strength of samples with various surface treatments at Cu-epoxy interfaces as a function of temperature cycles. The Cu-epoxy control sample without surface treatment shows the lowest peel strength through all 1500 temperature cycles, ranging from 0.4 to 1.4 N cm^−1^. Overall, peel strengths at the Cu-epoxy interfaces with various surface treatments followed this order: no treatment < azole < silane < Ni interlayer < multi-metal interlayer, even though this order fluctuates during temperature cycles. The Cu-azole-epoxy sample shows a continuous decrease in peel strength from 1.51 N cm^−1^ at 0 cycles to 0.46 N cm^−1^ at 1500 cycles, which is slightly higher than the Cu-epoxy sample. Silane coating exhibits improved peel strength, ranging from 0.7 to 2.1 N cm^−1^, compared to the Cu-epoxy and Cu-azole-epoxy samples. The Cu-epoxy and Cu-silane-epoxy samples show an increase in peel strength after the first 100 cycles, followed by a continuous decrease for the rest of the cycles. This trend can be due to improved interfaces associated with Cu(I) oxide, which is emphasized in our previous report.^30^ Silane coating further enhances the adhesion of Cu and epoxy via the formation of Cu-O-Si bonds.^30^ Thin Cu(I) oxides (< 100 nm) are advantageous for adhesion between Cu and epoxy, whereas thicker Cu oxides reduce peel strength due to their brittle nature.^30^

The interface with Cu-Ni-epoxy shows a constant decrease in adhesion strength from 2.1 N at 0 cycles to 0.9 N cm^−1^ at 1500 cycles. After 700 temperature cycles, Cu-Ni-epoxy exhibits higher peel strength than samples without metal interlayers, such as the Cu-epoxy, Cu-azole-epoxy, and Cu-silane-epoxy samples; however, it shows lower peel strength compared to other samples with multi-metal interlayers, such as NiCo_0.64_P_0.25_, NiW_0.48_P_0.10_, and CoW_0.66_P_0.17_. The NiCo_0.64_P_0.25_ and NiW_0.48_P_0.10_ samples exhibit a comparable level of peel strength. NiCo_0.64_P_0.25_ reduces the peel strength from 2.1 N cm^−1^ to 0.9 N cm^−1^ after 1500 temperature cycles. The peel strength of NiW_0.48_P_0.10_ varies from 2.3 to 0.9 N cm^−1^. The Cu-CoW_0.66_P_0.17_-epoxy sample exhibits the greatest overall peel strength, ranging from 2.6 to 1.1 N cm^−1^ throughout 1500 cycles. CoW_0.66_P_0.17_ and NiW_0.48_P_0.10_-plated samples show a slight increase in peel strength after 100 cycles, possibly due to stable W oxides.^25^ As shown in Fig. 6, the relatively high peel strength of metal interlayers, especially CoW_0.66_P_0.17_, is associated with mitigated oxide growth at Cu-epoxy interfaces. CoWP is reported to improve the oxidation resistance by forming a thin passivation oxide layer that mitigates O diffusion to the Cu layer.^25,52–54^

### Four-point probe

**Figure 7.**
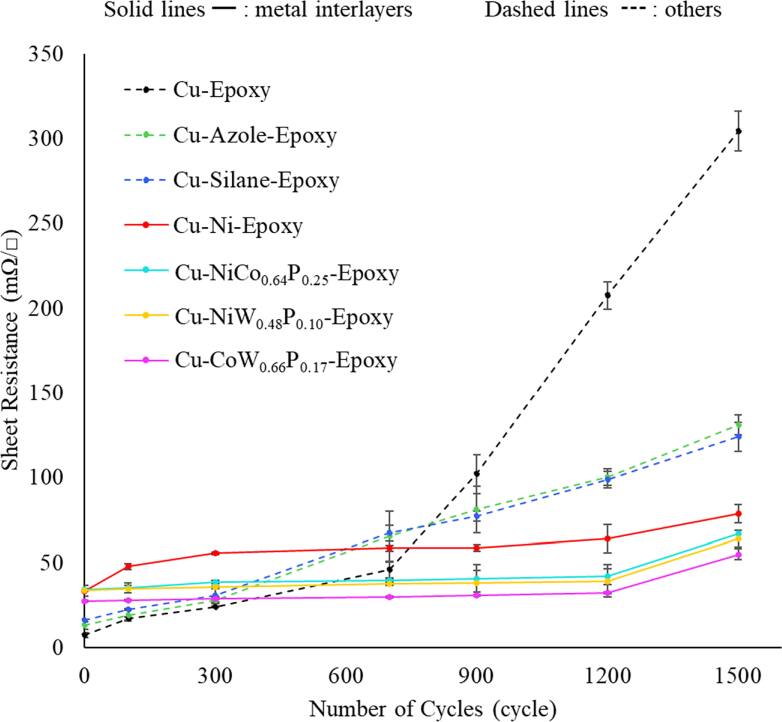
Sheet resistances of Cu-epoxy samples with azole coating, silane coating, Ni electroplating, and electroless plating of NiCo_0.64_P_0.25_, NiW_0.48_P_0.10_, and CoW_0.66_P_0.17_ after TCT. Solid lines represent metal interlayers, and dashed lines indicate the others. Error bars are included for all samples.

Figure 7 presents the effect of temperature cycling on sheet resistance. The sheet resistance was measured at the Cu surface after TCT. Overall, the sheet resistance increases with temperature cycles for all the samples. However, a rapid increase in the sheet resistance after 700 cycles is seen for the Cu-epoxy control sample, while for all the other samples, the increase is moderate.

The nucleation and growth mechanism of Cu oxides relates to the failure of Cu-epoxy systems. The Cu oxidation process involves nucleation (islands) and their subsequent growth, which results in the formation of a thin, continuous layer of Cu oxides.^55^ While nano and microscopic nucleation have a relatively low impact on larger-scale surface resistance measurement, the formation of continuous Cu oxide thin films has a greater effect on the measurements, leading to a rapid increase in sheet resistance and a decrease in peel strength.^30,55,56^ The Cu-epoxy shows a rapid increase in sheet resistance after 700 cycles. This is likely associated with significant oxide growth in the Cu-epoxy control sample.^57^ Azole coordination compounds are known to chemisorb on Cu surfaces through their lone-pair electrons, mitigating corrosion and reducing the associated increase in sheet resistance.^18–21,26^ The Cu-azole-epoxy and Cu-silane-epoxy samples show relatively lower rates of increase in sheet resistances; the Cu-azole-epoxy sample shows an increase in sheet resistance from 13.0 to 131.0 mΩ/☐, and the Cu-silane-epoxy sample rises from 16 to 125 mΩ/☐ over the 1500 cycles.

Samples with metal interlayers show relatively higher initial sheet resistance. The differences in absolute values of sheet resistance are due to the thin coating of the protective layer with higher resistivity and the surface-sensitive nature of the 4-point probe;^58^ however, samples with metal interlayers present lower sheet resistance than the other samples after 1500 cycles. The Ni-plated sample shows stabilized sheet resistance from 300 to 900 cycles, with values of 55.6 to 58.8 mΩ/☐, while it increases sheet resistance to 78.8 mΩ/☐ after 1500 cycles. The Cu-NiCo_0.64_P_0.25_-epoxy sample exhibits relatively stable sheet resistance ranging from 34.2 to 67.0 mΩ/☐. This stable sheet resistance range is comparable to Cu-NiW_0.48_P_0.10_-epoxy (33.7 to 64.1 mΩ/☐). The CoW_0.66_P_0.17_ sample shows the lowest sheet resistance compared to the other metal interlayers (27.3 to 54.9 mΩ/). The relatively low sheet resistance of metal interlayers is associated with mitigated oxide growth at Cu-epoxy interfaces,^57^ which is discussed further in the section titled “O Diffusion after TCT.” The long-term stability of sheet resistance can be compared as follows: Cu-CoW_0.66_P_0.17_-epoxy > Cu-NiCo_0.64_P_0.25_-epoxy and Cu-NiW_0.48_P_0.10_-epoxy > Cu-Ni-epoxy > Cu-azole-epoxy and Cu-silane-epoxy > Cu-epoxy.

### VNA

**Figure 8.**
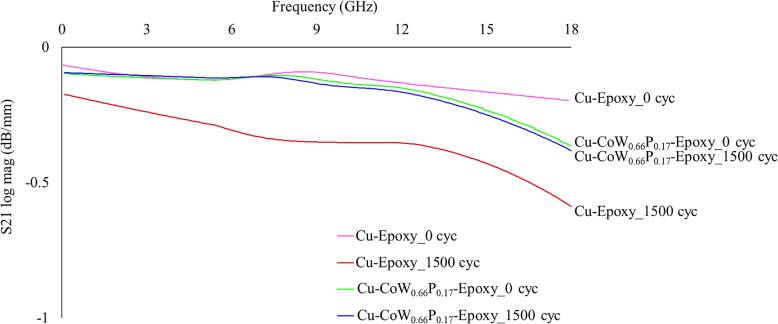
The impact of CoW_0.66_P_0.17_ interlayer on the insertion loss at the Cu-epoxy interface after 1500 temperature cycles as a function of frequency.

Further investigation using VNA was conducted for the sample with CoW_0.66_P_0.17_ interlayer, having the most stable sheet resistance and peel strength. Figure 8 shows the impact of CoW_0.66_P_0.17_ on the insertion loss of the Cu-epoxy system. The insertion loss trend for Cu conductors with an epoxy dielectric (without cycling) is similar to that reported in our previous publication.^43^ The transmission line with CoW_0.66_P_0.17_ shows stable insertion losses after TCT, showing only a 0.02 dB mm^−1^ difference at 18 GHz after 1500 temperature cycles. The Cu-epoxy control sample exhibits a 0.39 dB mm^−1^ difference at 18 GHz after 1500 cycles, which is approximately 20 times more than that of the Cu-CoW_0.66_P_0.17_-epoxy sample. The Cu-CoW_0.66_P_0.17_-epoxy sample exhibits relatively stable insertion loss from 0 to 10 GHz, with less than 0.06 dB mm^−1^ change regardless of temperature cycling; however, higher frequency results in exacerbated losses, with a ∼0.24 dB mm^−1^ change as the frequency increases from 10 GHz to 18 GHz, which are associated with the higher resistance and skin effect.^14,37^ The protective CoW_0.66_P_0.17_ layer between the Cu and epoxy increases resistance; however, metal interlayers exhibit relatively low insertion loss after cycling tests due to mitigated oxide growth at Cu-epoxy interfaces,^59^ which is discussed further in the section titled “O Diffusion after TCT.”

### O diffusion after TCT

**Figure 9.**
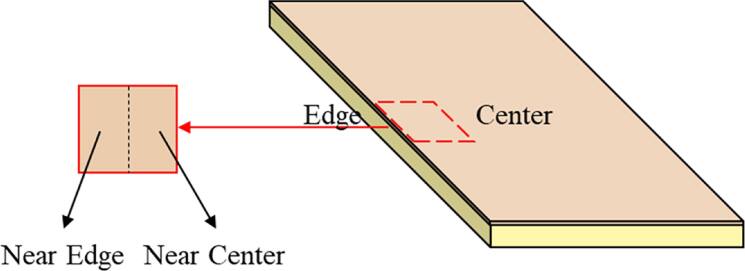
Schematic image illustrating the sample preparation process for EDS-SEM analysis. EDS-SEM results were obtained from the Cu side of the Cu-Epoxy interface.

Figure 9 shows the sample preparation for EDS-SEM analysis. Samples for EDS mapping of O were obtained from the edge of the Cu layer to observe O diffusion across the edge. EDS mapping was conducted from the Cu side of the Cu-epoxy interface to observe the oxidation of the Cu layer. EDS mapping illustrates the impact of surface treatments on O diffusion.

**Figure 10.**
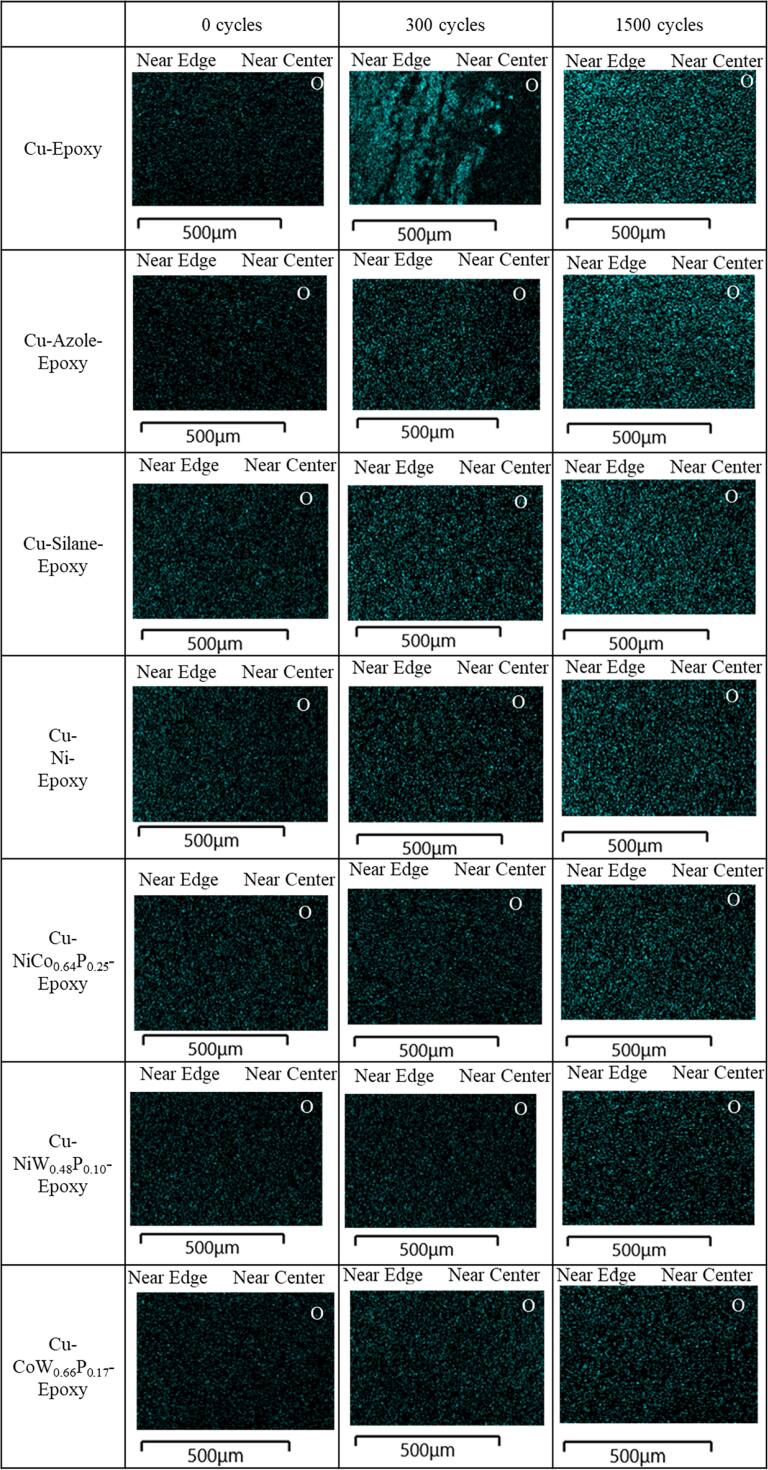
EDS mapping of Cu surfaces showing O diffusion at the Cu-epoxy interface with interlayers including methylimidazole, amine functional silane, and metal films such as Ni, NiCo_0.64_P_0.25_, NiW_0.48_P_0.10_, and CoW_0.66_P_0.17_.

Figure 10 shows a comparison of O diffusion at the Cu-epoxy interface with various adhesion promotion methods after 1500 temperature cycles. Figures S4–S10 show the additional images of EDS-SEM mapping of the samples. All samples show comparably low O on Cu surfaces at 0 temperature cycles. The Cu-epoxy control sample shows relatively aggressive O diffusion from the edge at 300 cycles. The Cu-epoxy sample at 1500 cycles shows that O is diffused more toward the center than at 300 cycles. At 300 cycles, the Cu-azole-epoxy presents more O at the edge than near the center. After 1500 temperature cycles, O is completely diffused from the edge toward the center of the Cu-azole-epoxy sample, showing a similar O composition at 1500 cycles compared to the Cu-epoxy sample. The Cu-silane-epoxy sample shows a higher O composition at 300 cycles compared to 0 cycles. At 1500 cycles, the Cu-silane-epoxy exhibits more diffused O; however, it has less O near the center compared to the Cu-azole-epoxy sample. In contrast, the Cu-epoxy interfaces with NiCo_0.64_P_0.25_, NiW_0.48_P_0.10_, and CoW_0.66_P_0.17_ interlayers show lower O composition after 300 and 1500 temperature cycles than the other samples. The metal interlayer at the Cu-epoxy interface suppresses significant oxide growth. Metal interlayers, especially CoW_0.66_P_0.17_, are reported to improve the oxidation resistance by forming a thin passivation oxide layer.^25,52–54^ The O diffusion results are associated with the stability of peel strength (Fig. 6) and sheet resistance (Fig. 7), exhibiting that more O diffusion correlates with lower peel strength and higher sheet resistance.

## Conclusions

These results show the cycling stability of Cu-epoxy interfaces with organic adhesion promoters, such as methylimidazole and amine functional silanes, and metal interlayers like Ni, NiCo_0.64_P_0.25_, NiW_0.48_P_0.10_, and CoW_0.66_P_0.17_. The results suggest that both organic and metal adhesion promoters improve the mechanical reliability of Cu-epoxy interfaces; however, the Cu-epoxy interfaces ultimately degrade with continued oxidation. The electroplated and electroless metal interlayers show greater stability than the organics based on the 90° peel test, four-point probe, and VNA results. Among the approaches we considered, the Cu-electroless CoW_0.66_P_0.17_-epoxy interface showed the best adhesion improvement with stable adhesion strength and sheet resistance after 1500 cycles. Both effects are likely due to their relatively stable oxides; however, this was at the penalty of increased conductor resistance. Of course, fully encapsulated dies and sealed packages further mitigate oxidant transport and limit the rate of oxidation; thus, the optimal approach depends on the application and desired reliability targets.
